# Structural characterisation of the fatty acid biosynthesis enzyme FabF from the pathogen *Listeria monocytogenes*

**DOI:** 10.1038/srep39277

**Published:** 2017-01-03

**Authors:** Tatiana P. Soares da Costa, Jeffrey D. Nanson, Jade K. Forwood

**Affiliations:** 1 School of Biomedical Sciences, Charles Sturt University, Wagga Wagga, New South Wales, 2678, Australia; 2 Department of Biochemistry and Genetics, La Trobe Institute for Molecular Science, La Trobe University, Melbourne, Victoria, 3086, Australia; 3 School of Chemistry and Molecular Biosciences and Institute for Molecular Bioscience (Division of Chemistry and Structural Biology) and Australian Infectious Diseases Research Centre, University of Queensland, Brisbane, 4072, Australia

## Abstract

Development of new antimicrobial agents is required against the causative agent for listeriosis, *Listeria monocytogenes*, as the number of drug resistant strains continues to increase. A promising target is the β-ketoacyl-acyl carrier protein synthase FabF, which participates in the catalysis of fatty acid synthesis and elongation, and is required for the production of phospholipid membranes, lipoproteins, and lipopolysaccharides. In this study, we report the 1.35 Å crystal structure of FabF from *L. monocytogenes*, providing an excellent platform for the rational design of novel inhibitors. By comparing the structure of *L. monocytogenes* FabF with other published bacterial FabF structures in complex with known inhibitors and substrates, we highlight conformational changes within the active site, which will need to be accounted for during drug design and virtual screening studies. This high-resolution structure of FabF represents an important step in the development of new classes of antimicrobial agents targeting FabF for the treatment of listeriosis.

*Listeria monocytogenes* is a Gram-positive intracellular bacterial pathogen that is the causative agent of listeriosis^1^. The unique ability of *L. monocytogenes* to cross several tight barriers within the infected human host contributes to its pathogenesis, leading to high hospitalisation and fatality rates^1^,^2^. This food-borne pathogen is especially dangerous in pregnant women, newborns, and the immunocompromised^3^, with current treatments for listeriosis generally consisting of a combination of supportive and antibiotic therapies. The first antibiotic resistant strains of *L. monocytogenes* were reported in 1988^4^. Since then, there has been a rapid increase in antibiotic resistant strains, mainly caused by self-transferable plasmids, mobilisable plasmids, and conjugative transposons^5^. Additionally, drug resistance conferred by bacterial efflux pumps has also been reported in *L. monocytogenes*^6^. As a result, there is an urgent need to develop novel classes of antibiotics against *Listeria spp.*, with new mechanisms of action.

One potential antibiotic target is the fatty acid synthesis (FAS) pathway, due to its essential role in the synthesis of phospholipid membranes, lipoproteins, and lipopolysaccharides^7^,^8^,^9^,^10^. There are two types of FAS pathways, FASI and FASII; the FASI pathway, found in animals and lower eukaryotes, encodes for a multifunctional complex, which is involved in fatty acid production and elongation^7^,^8^,^11^,^12^. Conversely, in the FASII pathway, found in bacteria, plants and parasites, each enzyme of the pathway is encoded by a separate gene that encodes for one protein, responsible for the catalysis of a single enzymatic step^7^,^8^.

In *Listeria spp*., the first FASII pathway reaction involves the condensation of fatty acid thioesters of varying acyl chain lengths and malonyl-acyl carrier protein (ACP). The initial condensation reaction of the FASII pathway involves short chain fatty acid thioesters (of one to four carbons in length), typically acetyl-CoA and malonyl-ACP, and is catalysed by β-ketoacyl-ACP synthase III (FabH), with subsequent rounds of condensation and further acyl chain elongation catalysed by β-ketoacyl-ACP synthase II (FabF). The condensation reactions catalysed by FabH and FabF result in the formation of β-ketoacyl-ACP products. These products are reduced by β-ketoacyl-ACP reductase (FabG) in an NADPH-dependent reaction to form β-hydroxyacyl-ACP. This in turn is dehydrated by β-hydroxyacyl-ACP dehydratase (FabZ) to form enoyl-ACP, before finally being reduced to acyl-ACP by the NADH-dependent enoyl-ACP reductase FabI to complete the cycle^8^. Given the essentiality of the FASII pathway in bacteria, as well as the structural differences between the mammalian and bacterial pathways, inhibitors of the FASII enzymes represent an important new class of antimicrobials with little to no mammalian toxicity^10^,^11^. The structural investigation of these enzymes is crucial to provide an enhanced understanding regarding the differences between pathogens and hosts, and is a valuable platform to guide the development of novel classes of antibacterial agents.

There are currently two commercially available antibiotics that target the FASII pathway, the broad-spectrum antiseptic triclosan and the anti-*Mycobacterium tuberculosis* agent isoniazid, both of which inhibit the enzyme catalysing the final reaction of the fatty acid elongation pathway (FabI)^13^,^14^. Additional FabI inhibitors, currently undergoing clinical trials, such as the triclosan derivatives AFN-1252 and MUT056399, display promising pharmacokinetics, pharmacodynamics, and efficacy *in vivo* against drug-resistant *Staphylococcus aureus* strains^15^,^16^,^17^,^18^, further demonstrating the validity of bacterial FASII pathways as an antibacterial target. The elongation enzyme FabF has also been shown to be an essential enzyme in the FASII pathway of *Bacillus subtilis*, which like *L. monocytogenes*, appears to utilise FabF as the sole β-ketoacyl-ACP synthase for elongation of medium to long chain fatty acids^19^. There are a number of known inhibitors that selectively inhibit FabF, including platensimycin^20^, cerulenin^21^,^22^,^23^, and thiolactomycin and its analogues^22^,^24^,^25^, as well as platensin^26^,^27^ that inhibits both FabF and FabH. However, none of these FabF inhibitors have been approved for use in the clinic, due to their poor pharmacokinetic properties, availability, and antimicrobial activity.

In this study, we describe the cloning, expression, purification, and high-resolution crystal structure of FabF from *L. monocytogenes* (*Lm*FabF). The characterisation of these structures is crucial for providing insights into the rational design of new classes of antimicrobial agents targeting FabF for the treatment of listeriosis.

## Materials and Methods

### Cloning

The gene encoding *Lm*FabF was amplified from genomic DNA (ATCC^®^ no. 19115D-5) by PCR using HotStarTaq PCR Master Mix (Qiagen) and primers 5′-ATGGATAAGAGAAGAGTAGTTGTTAC-3′ (forward) and 5′-TTAGTCTTCTATTCTTTTAAATACTAAAGTC-3′ (reverse). The PCR product was cloned into the expression vector pMCSG21 via ligation-independent cloning as previously described^28^,^29^,^30^, and confirmed by sequencing. To increase cloning efficiency, the T4 treated products were annealed using a 5:1 ratio of insert to vector, and the ligation time was increased to 1 hour, prior to transformation into chemically competent *Escherichia coli* TOP10 cells (Invitrogen). The final construct encoded the full length protein fused to an N-terminal hexahistidine (6-His) tag with a *Tobacco etch* virus (TEV) protease cleavage site for tag removal.

### Expression and purification

Recombinant *Lm*FabF was expressed as a His-tagged fusion protein in *E. coli* BL21 (DE3) pLysS competent cells. An 8 mL Luria Bertani (LB) starter culture supplemented with spectinomycin (100 μg mL^−1^) was used to inoculate 500 mL auto-induction medium^31^ supplemented with spectinomycin (100 μg mL^−1^), prior to incubation for ~16 h at 298 K with shaking until an OD_600_ of 3–4 was reached. Cultures were harvested by centrifugation at ~7,500 g for 30 min at 277 K. The resulting pellet was resuspended in buffer A (50 mM phosphate buffer pH 8.0, 300 mM sodium chloride, 20 mM imidazole) to a final volume of 30 mL, and frozen at 253 K. Lysozyme (1 mL, 20 mg mL^−1^) and DNAse (10 μL, 50 mg mL^−1^) were added to the thawed cell pellet at 273 K for 30 min. The resulting lysate was centrifuged at 12,000 g for 30 min at 277 K. The supernatant containing soluble *Lm*FabF was passed over Ni-NTA resin (HisTrap HP, GE Healthcare) pre-equilibrated with buffer A. The protein was eluted with an increasing concentration of buffer B (50 mM phosphate buffer pH 8.0, 300 mM sodium chloride, 500 mM imidazole). Fractions containing recombinant *Lm*FabF were incubated with TEV protease (0.2 mg mL^−1^) for 14 h at 277 K to cleave the His-tag. *Lm*FabF was further purified on a Superdex 200 column (GE Healthcare) equilibrated with buffer C (50 mM Tris pH 8.0, 125 mM sodium chloride). The purity of the protein was assessed to be greater than 95% using SDS-PAGE and concentrated to 20 mg mL^−1^ using a 10 kDa molecular weight cut-off Amicon ultracentrifugal device (Millipore), before storage at 193 K.

### Crystallisation

Initial crystallisation screens were performed using the following commercially available crystal screen kits: Crystal Screen 1 and 2, PEG/Ion 1 and 2 (Hampton Research), PACT Premier 1 and 2, and Proplex 1 and 2 (Molecular Dimensions). Crystal screens were performed using the hanging drop vapour diffusion method, where 1.5 μL recombinant *Lm*FabF solution was mixed with 1.5 μL reservoir solution on a siliconised cover slip, suspended over 300 μL reservoir solution, and incubated at 296 K. *Lm*FabF crystals were obtained in only one condition; Hampton Research Crystal Screen 2 condition 14 (0.2 M potassium sodium tartrate tetrahydrate, 0.1 M sodium citrate tribasic dihydrate pH 5.6, 2 M ammonium sulphate). After three days, large diffraction-quality crystals were obtained in 0.2 M potassium sodium tartrate tetrahydrate, 0.1 M sodium citrate tribasic dihydrate pH 5.6, 2 M ammonium sulphate, 22.5% glycerol, using a protein concentration of 20 mg mL^−1^.

### Data collection and structure determination

*Lm*FabF crystals were harvested after seven days and flash-cooled in liquid nitrogen at 100 K. Diffraction data were collected at the Australian Synchrotron on the MX2 beamline. A total of 160° of data were collected at 0.5° oscillations, a wavelength of 0.9537 Å, and a crystal-to-detector distance of 139.7 mm. Collected diffraction data were indexed, integrated, and scaled using *iMosflm*^32^ and *Aimless* from the CCP4 suite^33^,^34^,^35^. The structure of *Lm*FabF was solved by molecular replacement performed by *Phaser*^36^, using a monomer of the unpublished *L. monocytogenes* FabF structure as the search model (PDB ID: 3O04, RMSD between Cα atoms: 0.13 Å). Successive rounds of model building and refinement were performed using Coot^37^ and phenix.refine^38^,^39^.

The quaternary structure of *Lm*FabF and protein interfaces were investigated using the Protein Interfaces, Surfaces, and Assemblies’ service (PISA)^40^. Sequence alignments were generated using the T-Coffee (http://tcoffee.crg.cat/)^41^,^42^ and ESPript (http://espript.ibcp.fr/)^43^ web services. A summary of the crystallographic and refinement statistics are provided in Table 1.

## Results and Discussion

### Overall structure of *Lm*FabF

*Lm*FabF was successfully cloned and expressed in *E. coli*. Homogenous protein was obtained after purification by Ni-NTA and size exclusion chromatography, yielding pure monodisperse protein. Size exclusion chromatography suggested *Lm*FabF forms a dimer in solution, eluting as a product with a molecular weight of approximately 80 kDa. The purified *Lm*FabF showed a single band on SDS-PAGE, with an apparent monomeric molecular weight of 44 kDa, consistent with that of the theoretical molecular weight of the protein. Initial crystallisation attempts were performed using commercially available kits. Optimised crystals appeared after three days and were flash cooled on day seven, before data were collected using the MX2 microcrystallography beamline at the Australian Synchrotron. Images were integrated, merged, and scaled to a resolution of 1.35 Å. A total of 492,530 measured reflections were merged into 103,454 unique reflections, with an R_meas_ of 0.093. The crystal belongs to the space group P4_3_2_1_2, with unit cell parameters *a* = 73.75, *b* = 73.75, *c* = 170.27. A single *Lm*FabF molecule was placed in the asymmetric unit, giving a Matthew’s coefficient of 2.69 Å^3^ Da^−1^ and a solvent content of 54%. The final refined structure of *Lm*FabF displayed R_work_ and R_free_ values of 0.148 and 0.165, respectively. A summary of the crystallographic and refinement statistics are provided in Table 1.

**Figure 1:**
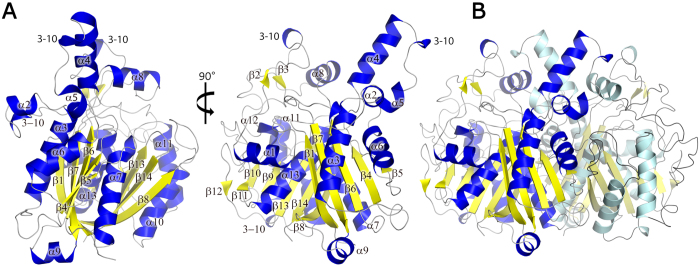
An overview of the structure of *Listeria monocytogenes* FabF (*Lm*FabF), showing a cartoon representation of the *Lm*FabF subunit at 0° and 90° of rotation around the horizontal axis (**A**), as well as the *Lm*FabF dimer (**B**). β-sheets are displayed in yellow; α-helices are displayed in blue. The core of the *Lm*FabF structure is comprised of a five-layered thiolase fold motif, with two five-stranded mixed β-sheets flanked either side by two α-helices.

The *Lm*FabF monomer within the asymmetric unit contains 13 α-helices and 14 β-strands, which are predominantly arranged in a central motif. The core motif of *Lm*FabF and similar homologues appears to adopt a thiolase type fold, which is characteristic of the FASII condensing enzymes^44^,^45^,^46^,^47^,^48^. This core motif consists of two, five-stranded mixed β-sheets flanked either side by two α-helices, arranged into a five-layered α-β-α-β-α topology in which each α represents two α-helices and each β represents a five-stranded mixed β-sheet (Fig. 1). Despite the presence of a single *Lm*FabF monomer within the asymmetric unit, tight packing of molecules related by crystallographic symmetry indicates that *Lm*FabF forms a dimer in solution. Analysis of the crystal structure of *Lm*FabF using PISA also suggests that *Lm*FabF forms a dimer in solution, which is consistent with our size exclusion chromatography data as well as bacterial homologues, including *E. coli, B. subtilis, Streptococcus pneumoniae,* and *Yersinia pestis,* that also appear to form similar dimers in solution^29^,^44^,^48^,^49^.

**Figure 2:**
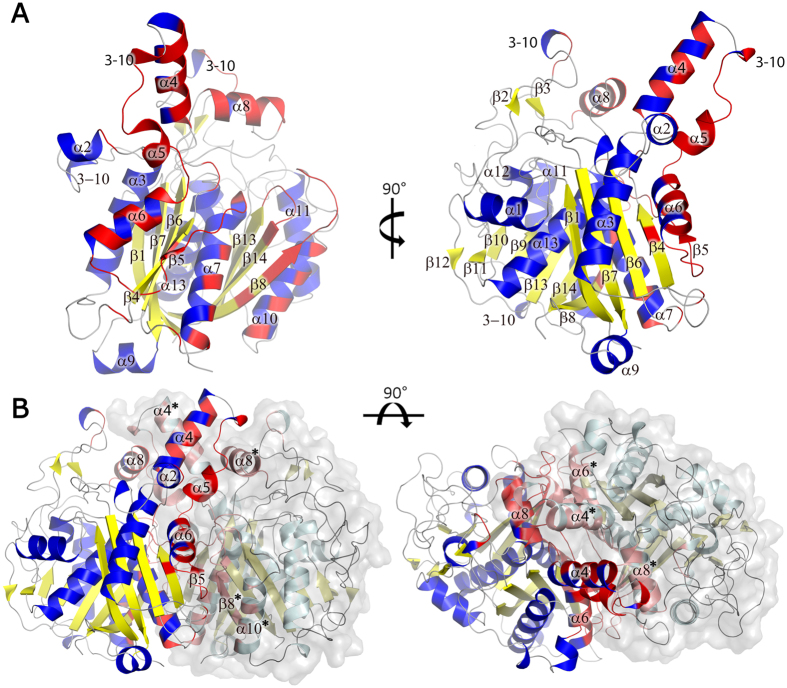
A cartoon representation of the *Lm*FabF dimer interfaces. (**A**) An *Lm*FabF monomer at 0° and 90° of rotation around the horizontal axis, with residues forming the dimer interface displayed in red. (**B**) *Lm*FabF monomers of the dimeric assembly align anti-parallel at the edge of the thiolase fold, with the loops connecting sheet β5 and helix α7 of each monomer packing against their counterparts on the opposing monomer. Helices α4 of each monomer pack against each other. Helices α4 and α5 also pack against α8 of the opposing subunit. Additional interactions are formed between α6, β5, α7 and the connecting loop regions with the loop regions between β8 and α10, and β13 and β14 of the opposing monomer. Interfacing residues are displayed in red; interfacing residues of the opposing monomer are displayed in pink. Labelled secondary structure features of the opposing monomer are indicated by asterisks (*).

The *Lm*FabF monomers of the dimeric assembly are related by a crystallographic two-fold axis, with the loop regions connecting strand β5 and helix α7 of each monomer packing against their counterparts on the opposing monomer in an anti-parallel fashion. Helices α4 of each monomer also pack against each other, and helices α4 and α5 pack against α8 of the opposing subunit. Additional interactions are formed between α6, β5, α7 (residues ~140–180) and two loop regions connecting β8-α10 (residues ~260–270) and β13-β14 (residues ~398–402) of the opposing monomer. These regions form several hydrogen bonds and two salt bridges, with approximately 18% (~2,875 Å^2^) of the total solvent accessible surface area buried by the dimer interface, and a solvation free energy gain of ~205 kJ mol^−1^ upon dimer formation (Fig. 2).

### The *Lm*FabF active site and reaction mechanism

**Figure 3:**
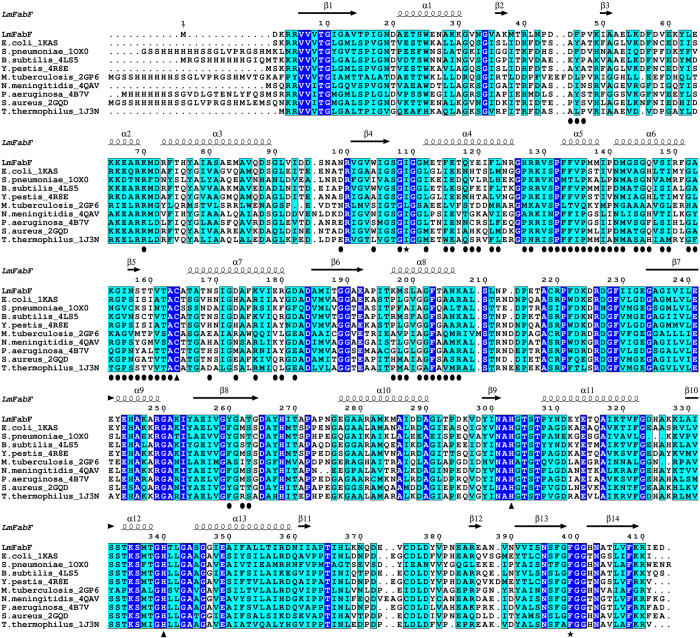
The catalytic triad and surrounding residues of FabF enzymes are highly conserved, as evident in the sequence alignment of *Lm*FabF with FabF from *E. coli* (PDB: 2GDW), *S. pneumoniae* (PDB: 1OX0), *B. subtilis* (PDB: 4LS5), *Y. pestis* (PDB: 4R8E), *M. tuberculosis* (PDB: 2GP6), *N. meningitidis* (PDB: 4QAV), *P. aeruginosa* (PDB: 4B7V), *S. aureus* (PDB: 2GQD), and *T. thermophilus* (PDB: 1J3N). Strictly conserved residues are highlighted in blue with white text; similar residues are highlighted in cyan; residues of the active site catalytic triad are designated by triangles; residues Ile109 and Phe399, which undergo conformational changes in order to accommodate binding of substrates and inhibitors, are designated by a black star. The α-helices and β-sheets of *Lm*FabF are depicted above their corresponding residues. Interfacing residues of the *Lm*FabF dimer are designated by a black circle.

The reaction mechanism and active sites of FabF enzymes have been well characterised in bacterial homologues, such as *E. coli* and *S. pneumoniae*. The central Cys/His/His active site is comprised of Cys164, His303, and His340 in *Lm*FabF, and is positioned at the junction of two substrate-binding pockets (Fig. 3). The condensation reaction catalysed by FabF enzymes is thought to occur by a ping-pong mechanism. In the first step, an acyl group of a fatty acyl donor (typically an acyl-ACP) is transferred to the active site cysteine (Cys164 in *Lm*FabF) by nucleophilic attack, and the carrier molecule (ACP) of the bound fatty acyl donor molecule dissociates. In the second step, the fatty acyl thioester to be elongated (typically malonyl-ACP) binds, and a second nucleophilic attack induces the transfer of the acyl group from the condensing enzyme to the recipient, forming a β-ketoacyl-ACP product. The histidine residues His303 and His340 of the catalytic triad are thought to be involved in inducing the second nucleophilic attack of the catalytic mechanism and forming an oxyanion hole to stabilise the fatty acyl-enzyme intermediate during transition states^22^,^44^,^48^,^50^.

**Figure 4:**
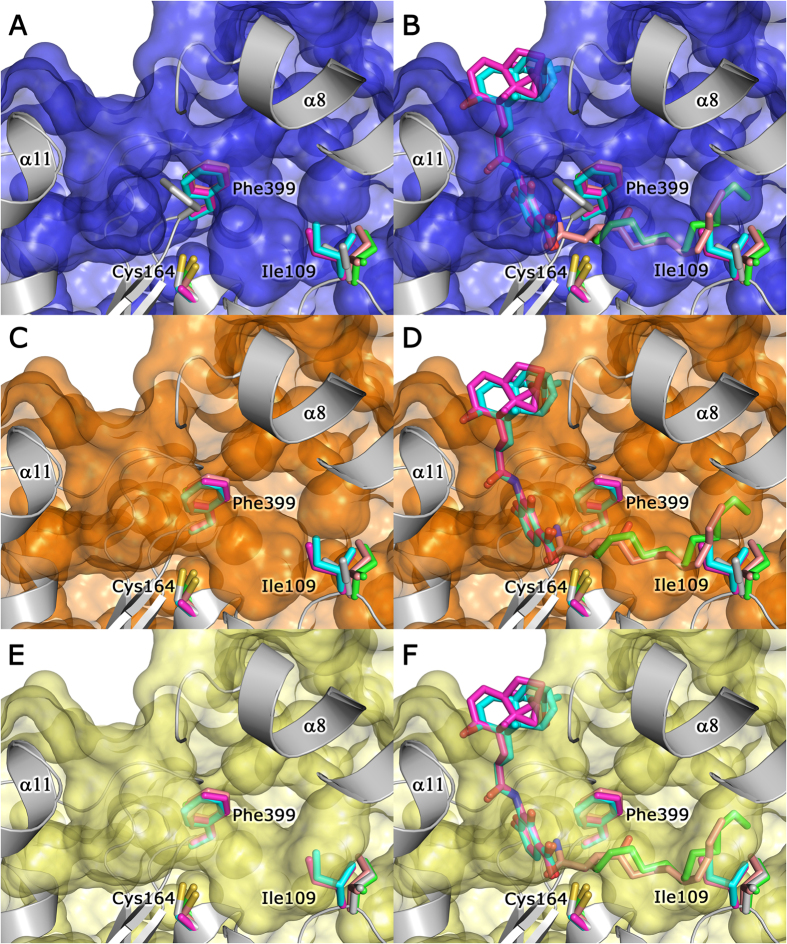
The *Lm*FabF substrate binding pocket appears partially closed, and must undergo conformational changes in order to accommodate binding of substrates and inhibitors. Superposition of the structures of *E. coli* FabF (*Ec*FabF) in complex with cerulenin (PDB: 1B3N, pink), platencin (PDB: 3HO2, magenta), platensimycin (PDB: 3HNZ, cyan), and dodecanoic acid (PDB: 2GFY, green) shows that Phe399 and Ile109 of *Lm*FabF (silver) partially close the substrate binding pocket (shown as a surface model) (**A**), clashing with cerulenin and dodecanoic acid (**B**). The binding pocket appears partially opened upon rotation of Phe399 (silver sidechain) to a conformation almost identical to that observed in the structures of *Ec*FabF, overlapping with the phenylalanine sidechain of *Ec*FabF (**C**). However, *Lm*FabF is still unable to fully accommodate cerulenin and dodecanoic acid (**D**), with the additional rotation of Ile109 (silver sidechain) required to accommodate the entire acyl chain of these molecules (**E,F**). Note: platencin and platensimycin structures contain a Cys164Ala mutation.

We attempted to obtain structures of *Lm*FabF-ligand complexes through soaking at a 10:1 ligand to protein molar ratio for 2 hours and 24 hours. Our attempts to crystallise *Lm*FabF with known inhibitors and substrates via soaking were unsuccessful. However, due to the conserved nature of bacterial FabF proteins, we were able to compare the structure of *Lm*FabF to other bacterial homologues complexed with known inhibitors and substrates. These comparisons indicate no steric clashes between the inhibitors platencin and platensimycin (PDB IDs: 3HO2 and 3HNZ, respectively) with residues within the *Lm*FabF active site. However, the inhibitor cerulenin and the medium chain fatty acid dodecanoic acid (PDB IDs: 1B3N and 2GFY, respectively) both clash with the side-chains of Phe400 and Ile108 (Phe399 and Ile109 in *Lm*FabF), which lie within the substrate binding pocket. Comparison of the structures of *E. coli* FabF (*Ec*FabF) bound to cerulenin, platencin, platensimycin, and dodecanoic acid to that of *Lm*FabF clearly shows that the side chains of Phe400 and Ile108 adopt different conformations to accommodate these molecules (Fig. 4A,B).

### The *Lm*FabF substrate binding site is partially closed

During catalysis, access to the substrate binding pockets of FabF enzymes appears to be predominantly controlled by residues Phe400 and Ile108 (Phe399 and Ile109 in *Lm*FabF)^48^,^49^. In *Lm*FabF and other unliganded FabF structures, the side chain of Phe400 packs against the active site, partially closing the substrate binding site and preventing access to the nucleophilic Cys163 (Fig. 4A,B). Rotation of Phe399 in *Lm*FabF to a conformation similar to that observed in the structures of *Ec*FabF bound to cerulenin^23^, platencin, platensimycin^51^, and dodecanoic acid^20^ (PDB IDs: 1B3N, 3HO2, 3HNZ, and 2GFY, respectively) appears to partially open the substrate binding pocket, allowing access to the active site (Fig. 4C,D). Access to the full length of this binding pocket appears to be blocked by the conformation of Ile109, which clashes with both cerulenin and dodecanoic acid. However, these clashes can be resolved by rotating Ile109 in *Lm*FabF to a conformation similar to that observed in the structures of *Ec*FabF in complex with cerulenin and dodecanoic acid, which opens the binding pocket to accommodate the full length of these two molecules (Fig. 4E,F).

Mutation of Phe400 to alanine reveals the crucial role of Phe400 in determining the order of substrate binding and maintaining forward (condensation) reaction activity, with the rate of the reverse reaction observed to exceed the forward condensation reaction activity greatly in this mutant^48^. While Phe400 is clearly involved in controlling FabF substrate binding, the role of Ile109 (Ile108 in *Ec*FabF) is still unclear. The finding that Ile108Phe mutants in both *E. coli* and *B. subtilis* display reduced elongation of long-chain acyl-ACPs^49^, presumably mimicking the shortened binding pocket observed in the *Lm*FabF structure, suggests that Ile109 may play a role in determining substrate specificities for the long-chain acyl-ACPs. Price *et al*. (2001) suggested that Ile108 plays a role in directing the acyl chain of acyl-ACPs into the substrate binding pocket, and is at least partially accountable for the differences in substrate specificity and physiological function observed between FabF and FabB, which is able to catalyse the elongation of unsaturated fatty acid intermediates that are not elongated by FabF^22^. In the absence of further data, the role of Ile108/109 remains to be fully elucidated. The conformational changes required by Phe399 and Ile109 to accommodate substrates and inhibitors may be of particular importance for structure-based drug design or virtual screening of potential antimicrobial agents that target these regions of the *Lm*FabF substrate-binding pocket, however such studies should first take into consideration the flexibility of these residues to prevent erroneous interpretation of results.

## Conclusion

Here we report the 1.35 Å crystal structure of FabF from *L. monocytogenes*. The high-resolution structure of this enzyme provides an accurate platform for structure-based drug design and virtual screening of new antimicrobial agents. Comparison of *Lm*FabF with *Ec*FabF in complex with the inhibitors cerulenin, platencin, and platensimycin indicated no steric clashes that would prevent platencin or platensimycin from binding *Lm*FabF, and that cerulenin may be accommodated upon conformational changes that appear to occur within the substrate binding pocket during substrate/inhibitor binding. These conformational changes should be considered during drug design and virtual screening studies, as results may be interpreted erroneously if the flexibility of these residues is not first taken into account.

## Additional Information

**How to cite this article**: Soares da Costa, T. P. *et al*. Structural characterisation of the fatty acid biosynthesis enzyme FabF from the pathogen *Listeria monocytogenes. Sci. Rep.* **7**, 39277; doi: 10.1038/srep39277 (2017).

**Publisher's note:** Springer Nature remains neutral with regard to jurisdictional claims in published maps and institutional affiliations.

## Figures and Tables

**Table 1:** *Lm*FabF crystallographic and refinement statistics.

PDB ID	5SXO
Resolution range (Å)	1.35–28.52
Space group	P4_3_2_1_2
Unit cell length (Å)	a = 73.75, b =73.75, c = 170.27
Unit cell angle (°)	90
Total observations	492,530 (18,953)
Unique observations	103,454 (5,097)
Multiplicity	4.8 (3.7)
Completeness (%)	99.7 (100.0)
Mean I/sigma (I)	8.9 (2.6)
Mean CC (1/2)	0.997 (0.879)
R_pim_	0.031 (0.250)
R_meas_	0.093 (0.494)
R_merge_	0.087 (0.367)
R_work_	0.1479
R_free_	0.1653
Number of atoms	
Non-solvent*	3076
Solvent	372
RMSD bonds (Å)	0.003
RMSD angles (°)	0.637
Ramachandran favoured (%)	98
Ramachandran allowed (%)	2
Ramachandran outliers (%)	0
Rotamer outliers (%)	0.31
Clashscore	0.00
Average B-factor	22.48
Macromolecules	21.09
Solvent	34.01

^*^Note: Not including hydrogen atoms.
